# Implementation of physical examination pro forma – a complete audit cycle

**DOI:** 10.1192/bjo.2021.310

**Published:** 2021-06-18

**Authors:** Deshwinder Singh Sidhu, Guy Molyneux

**Affiliations:** 1 Phoenix Care Centre; 2 HSE St. Aloysius Mater Misericordiae University Hospital

## Abstract

**Aims:**

Aim of this audit is to achieve and maintain 100% compliance in physical examination on admission.

**Background:**

Conducting physical examination on admission is a mandatory requirement and is monitored by the Mental Health Commission during yearly inspections. A report published by Inspectorate of the Mental Health Commission recently in 2019 identifies a gap in physical health monitoring. We conducted a complete audit cycle in an inner city hospital psychiatric ward to monitor compliance with physical examination on admission.

**Method:**

We based the audit on Judgment Support Framework (JSF) version 5 standards. A retrospective review of all of the patient's medical records was carried out. 13 medical records were reviewed in the first cycle. The results of the first cycle were presented to the Multi Disciplinary Team (MDT) members, including the Non-Consultant Hospital Doctors (NCHD). Physical health policy was reviewed, in consultation with the committee and Clinical Director, a Physical Examination pro-forma (colour coded) was developed and implemented. It was based on the National Guidelines and the JSF ver.5. All members of the MDT and NCHDs were briefed on the pro forma introduced. A repeat audit cycle was conducted of all patients admitted after first audit cycle. Data were collected using a simple audit tool indicating if physical examination was conducted or refused.

**Result:**

A total of 22 medical records were audited. 13 medical records in the first cycle indicted only 3 patients had physical examination on admission. However, prior to admission a total of six patients had physical exam in the Emergency Department (ED). Upon implementation of the pro forma, 9 medical records of all patients admitted post-first cycle were audited. A total of 7 patients had physical examination on admission to the ward. Two patients refused physical examination and this was clearly documented. One patient had physical examination completed in ED. All newly admitted patients had physical examination completed or the reason why it wasn't completed documented clearly.

**Conclusion:**

Physical examination pro forma was successfully implemented, raising current compliance to a 100%, with a significant improvement from 23% compliance in the first cycle. Existing pro forma is helpful as a reminder to NCHDs. Colour coding of pro forma improves accessibility and distinguishability during the process of admission and auditing. Physical examination pro forma will be audited every 6 monthly.

